# Diagnostic change 2 years after a first episode of psychosis

**DOI:** 10.1192/j.eurpsy.2021.1416

**Published:** 2021-08-13

**Authors:** L. Aranguren, M. Martinez, E. García De Jalón, A. Fernández, M.C. Ariz, M. Otero, N. Pereda

**Affiliations:** 1 Salud Mental. Primeros Episodios, Servicio Navarro de Salud, Pamplona, Spain; 2 Mental Health Group, Instituto de Investigación Sanitaria de Navarra (IdISNA), Pamplona, Spain

**Keywords:** psychosis, stability, Diagnostic, consistency

## Abstract

**Introduction:**

Psychiatric diagnoses are derived from expert opinion (1). Since no objective tests or markers are on the horizon, clinical psychiatry is anchored to “the patient’s altered experience, expression and existence, associated with suffering in self and/or others”(2). Many studies have examined diagnostic stability over time. In the last years investigators have been reporting prospective and retrospective consistencies of diagnoses between two time points, specially in first episodes of psychosis (3).

**Objectives:**

To examine the prospective and retrospective stability of diagnostic categories 2 years after the first episode of psychosis

**Methods:**

Data were examined from the First Episode Psychosis Program of Navarra (PEPsNA), a prospective observational study of a cohort of patients with first-episode psychosis in Navarra (Spain). Diagnosis was assigned using DSM-IV-TR at baseline and 24 months later. Diagnoses were divided into 5 categories: Affective psychosis, Schizophrenia spectrum psychosis, Schizoaffective disorder, acute psychosis and other diagnoses. Diagnostic change was examined using prospective and retrospective consistency

**Results:**

A total of 78 first-episode psychosis cases with baseline and 24 months follow-up were identified. Table 1 shows the diagnosis movement matrix, and Figure 1 its graphical representation. Of cases, 71.8% (56/78) had the same baseline and 24 months follow-up diagnosis. Prospective and retrospective consistencies are shown in Table 2

**Conclusions:**

The prospective and retrospective consistencies of Schizophrenia spectrum psychosis and acute psychosis were higher than others. Affective psychosis and Schizoaffective disorder show very variable consistencies

